# Cardiac implications of hypoglycaemia in patients with diabetes – a systematic review

**DOI:** 10.1186/1475-2840-12-135

**Published:** 2013-09-21

**Authors:** Markolf Hanefeld, Eva Duetting, Peter Bramlage

**Affiliations:** 1 Study Centre Professor Hanefeld, GWT, TU Dresden, Dresden, Germany; 2 Novartis Pharma GmbH, Nürnberg, Germany; 3 Institut für Pharmakologie und präventive Medizin, Mahlow, Germany

**Keywords:** Hypoglycaemia, Cardiovascular risk, Arrhythmia, Continuous glucose monitoring, Randomized controlled trials

## Abstract

**Background:**

Hypoglycaemia has been associated with increased cardiovascular (CV) risk and mortality in a number of recent multicentre trials, but the mechanistic links driving this association remain ill defined. This review aims to summarize the available data on how hypoglycaemia may affect CV risk in patients with diabetes.

**Methods:**

This was a systematic review of available mechanistic and clinical studies on the relationship between hypoglycaemia and cardiovascular risk. Study outcomes were compiled from relevant articles, and factors contributing to hypoglycaemia-mediated CVD and its complications are discussed.

**Results:**

Six recent comprehensive clinical trials have reinforced the critical importance of understanding the link between hypoglycaemia and the CV system. In addition, 88 studies have indicated that hypoglycaemia mechanistically contributes to CV risk by increasing thrombotic tendency, causing abnormal cardiac repolarization, inducing inflammation, and contributing to the development of atherosclerosis. These hypoglycaemia-associated risk factors are conducive to events such as unstable angina, non-fatal and fatal myocardial infarction, sudden death, and stroke in patients with diabetes.

**Conclusions:**

Emerging data suggest that there is an impact of hypoglycaemia on CV function and mechanistic link is multifactorial. Further research will be needed to ascertain the full impact of hypoglycaemia on the CV system and its complications.

## Background

In patients with type 1 (T1D) and type 2 diabetes (T2D), cardiovascular (CV) disease is the most common cause of death (45% and 52%, respectively) [1-3]. Since diabetes is a disease of glucose intolerance, most studies have focused on elucidating the role of hyperglycaemia on CV complications. However, recent reports have also begun to highlight the potential importance of hypoglycaemia-mediated adverse effects [4-6].

Episodes of hypoglycaemia are frequent in diabetic patients undergoing intensive glucose lowering therapy. In fact, hypoglycaemia constitutes the principal reason that blood glucose targets are not achieved in many patients [7]. Interestingly, recent studies that specifically examined the benefits of intensive glucose lowering therapy such as the Action to Control Cardiovascular Risk in Diabetes (ACCORD) [8] trial, the Action in Diabetes and Vascular Disease: Preterax and Diamicron Modified Release Controlled Evaluation (ADVANCE) study [9] and the Veteran’s Affairs Diabetes Trial (VADT) [10], did not observe a reduction in CV risk. Instead, these trials demonstrated significantly increased rates of hypoglycaemia in the intensive treatment arms, which was implicated in the lack of a benefit and possibly even excess mortality in the ACCORD trial [8,11].

Because hypoglycaemia is often asymptomatic, it is difficult to establish a direct association with mortality. Nevertheless, efforts have been made to identify possible mechanistic links between hypoglycaemia and CV complications during diabetes treatment. So far, it has been suggested that acute or recurrent hypoglycaemic episodes could induce thrombosis and inflammation [12], abnormal cardiac repolarization, arrhythmia and atrial fibrillation [13], endothelial injury [14], myocardial ischemia and cerebral damage [15], and preclinical atherosclerosis [16]. In addition, a link has been made between low glucose levels and the unexpected sudden death of patients with type 1 diabetes without CVD, also known as “dead in bed” syndrome [17].

The effects of hypoglycaemia are poorly established and sometimes overlooked, but can have potentially life-threatening consequences [18]. Because of this, we aimed to review the available data regarding the impact of hypoglycaemia on cardiac complications and mortality in patients with diabetes.

## Methods

### Search strategy

An electronic literature search of PubMed up to November 2012 was performed using a combination of subject headings and free text incorporating “hypoglycaemia”, “cardiac OR heart”, “QTc”, “arrhythmia”, “endothelium”, “thrombosis”, “inflammation”, “atherosclerosis”, “heart rate”, “heart failure”, “myocardial ischemia”, “myocardial infarction”, “coronary heart disease”, and “angina”. The search was then extended by manually screening the reference lists of included papers.

### Study selection

Identified sources where evaluated according to the PRISMA statement for reporting systematic reviews and meta-analyses of studies [19]. Papers were first subdivided based on whether they were clinical trials involving glycaemic control or studies related to the underlying pathophysiologic mechanisms linking hypoglycaemia with CV risk. Included clinical studies fulfilled all of the following criteria: (1) published as a primary research paper in a peer-reviewed journal; (2) included cohorts of patients with T1D or T2D under intensive glucose-lowering therapy; and (3) reporting the proportion of patients presenting with CV complications. Studies of only highly selected groups (e.g. undergoing cardiac surgery or case reports), and conference proceedings were excluded. Included mechanistic studies fulfilled the following criteria: (1) published as a primary research paper in a peer-reviewed journal, and (2) investigated direct mechanistic links between hypoglycaemia-induced physiologic effects and CV risk. Only papers in English were selected. One reviewer performed the search and screened the titles and abstracts to exclude irrelevant papers. Full-text articles were then reviewed by at least two reviewers to assess eligibility, and consensus was sought to resolve any disagreement between researchers.

### Quality assessment

We applied the Critical Appraisal Skills Programme guidelines for quality assessment and excluded studies with major limitations in methods or reporting [20]. In addition, the quality of each included study was rated high, moderate or low according to pre-specified markers of quality (Table 1).

**Table 1:** Criteria of high, moderate and low study quality

**Study quality**	**Criteria**
High: small risk of bias	Prospective study design and the following:
	- Adequately described patients constituting a representative and clinically relevant sample
	- Sample size ≥ 5000
Moderate: moderate risk of bias	Prospective study design
Low: high risk of selection and/or verification bias	Retrospective study design
	Selected or enriched samples

### Data collection and analysis

Characteristics of included studies were extracted independently by two researchers using a standardised form. These included study design, population, treatments, and CV outcome. A qualitative analysis of the texts was performed.

## Results

### Definition and frequency of hypoglycaemia

#### Definition

There has been some debate over which biochemical definition of hypoglycaemia should be used in clinical settings [21]; however, the most well-known classification put forth by the American Diabetes Association (ADA) Workgroup on Hypoglycaemia [22] included criteria for five categories of hypoglycaemia: severe hypoglycaemia (requiring aid of another person to administer treatment), documented symptomatic hypoglycaemia (common hypoglycaemic symptoms and measured plasma glucose of ≤ 70 mg/dL [3.9 mmol/L]), asymptomatic hypoglycaemia (not accompanied by symptoms but glucose measurement of ≤ 70 mg/dL [3.9 mmol/L]), probable symptomatic hypoglycaemia (self-reported symptomatic episode not verified by glucose determination), and relative hypoglycaemia (symptoms associated with plasma glucose > 70 mg/dL [3.9 mmol/L]). Importantly, hypoglycaemia often occurs during sleep, and these episodes of “nocturnal hypoglycaemia” can range from asymptomatic to severe. From Continous Glucose Monitoring System (CGMS) studies it is known that the majority of critical low (< 3.1 mmol/l) glucose nocturnal episodes remain unrecognized [23].

Normally, when blood glucose levels fall below the threshold for supporting normal cognitive function, the body initiates glucose counter-regulatory measures, which include release of glucagon and epinephrine. However, an important response driven by recurrent hypoglycaemia in T1D and advanced T2D involves the impairment of physiological mechanisms that normally defend against declining plasma glucose levels. This is especially true for patients with T1D, who lose normal glucagon response to hypoglycaemia within five years of diagnosis and have attenuated epinephrine release [24]. This leads to the development of “hypoglycaemia unawareness”, which involves the loss of the hypoglycaemia-related symptoms that normally alert diabetic patients to take corrective action [25]. As a result, recurrent and unnoticed cycles of potentially dangerous hypoglycaemia can ensue, particularly in the case of insulin-requiring diabetes. Individuals with impaired hypoglycaemia awareness have a six-fold increased risk for experiencing severe hypoglycaemia [26], and attenuated physiological responses to critical low glucose levels might be fatal [27].

#### Frequency

Hypoglycaemia is a frequent finding resulting from inadaequate insulin dosing in T1D. In fact, patients with T1D experience thousands of mild (glucose < 70 mg/dL) hypoglycaemic episodes over a lifetime of diabetes, suffering on average two episodes of symptomatic hypoglycaemia per week, and at least one severe episode per year [28]. A 2007 study reported the incidence of severe hypoglycaemia to be 110 episodes per 100 patient-years in patients with T1D treated with insulin for <5 years and 320 episodes per 100 patient-years in those treated for >15 years with insulin [7]. Also, it has been suggested that 6-10% of deaths in patients with T1D could result from hypoglycaemia [29,30].

In the case of T2D, hypoglycaemia may be less frequent, and usually results from the use of drugs that cause increased endogenous insulin levels (e.g. sulfonylureas) or treatment with exogenous insulin [31-34]. If T2D patients were treated with insulin, the overall rate of hypoglycaemia was reported to be low [11] and about one third that of patients with T1D [35]; however, when considering advanced stages of T2D, the frequency approaches that of T1D [7]. It is particularly difficult in a frail elderly population which not only have a high risk for developing hypoglycaemia but also because of their hypoglycaemia associated autonomic failure [36].

### High quality clinical studies on glycaemic control and CV outcome in diabetes

**Figure 1:**
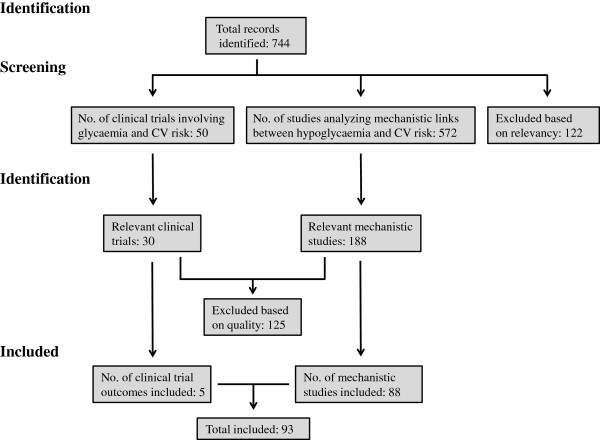
Flow diagram of the study identification process.

The most common cause of death in diabetes is CV disease (CVD) [1,2], and there exists a bulk of evidence that hypoglycaemia is a serious CV risk factor [4,5]. Six high quality studies have examined CV outcome while comparing intensive glucose-lowering treatment with conventional therapy (Figure 1 & Table 2).

**Table 2:** **Characteristics of high quality clinical trials identified (adapted from**[37,38]**)**

**Study**	**Population**	**Design**	**Treatments**	**Outcome**
DCCT/EDIC [39,40]	1,441 T1D adolescents and adults (13–39 years old) with diabetes duration of 1–15 years	Effect of intensive vs. conventional treatment on micro- and macrovascular complications	Intensive treatment (multiple injections or pump) vs. standard therapy	↓CVD by 54%, but only evident after long-term (>12-yr.) follow-up
UKPDS [41,42]	5,102 newly diagnosed T2D adults	Randomized control trial of intensive therapy to reduce complications of T2D	Two intensive treatment arms (insulin/sulfonylurea or metformin) vs. conventional therapy	No significant differences in CV outcomes after trial, but 10-yr. follow-up revealed a modest reduction in CVD
ACCORD [8,43,44]	10,251 T2D patients, 40–79 years of age with CV or 55–79 years of age with atherosclerosis or ≥ two risk factors	3.5 yr. study; Randomized control trial of excellent HbA1c (<6%) vs. 7.0–7.9%	Combinations of all available treatments to achieve goal HbA1c	Study stopped early because of increased overall and CV mortality; primary CVD endpoint ↓10% (P=0.16); overall mortality ↑22% (P=0.04); CV mortality ↑35% (P=0.02)
ADVANCE [9]	11,140 patients with T2D in 20 countries, ≥55 years of age and ≥30 years of age at diagnosis	5 yr. study; tested if glucose lowering affected CV risk in T2D patients with at least one risk factor	Intensive glucose lowering (≤6.5%) vs. standard treatment	No difference in CV end point by treatment group; primary CVD endpoint ↓6% (P=0.37); overall mortality ↓7% (P=NS); CV mortality ↓12% (P=NS)
VADT [10,45,46]	1,791 patients with T2D on insulin or maximal-dose oral agents	5.6 yr. study; determined effect of intensive glycaemic control on CV risk	Intensive treatment (<6.0%) vs. standard treatment	No difference in CV end point by treatment group; primary CVD endpoint ↓13% (P=0.12); overall mortality ↑6.5% (P=NS); CV mortality ↑25% (P=NS)
ORIGIN [47]	12,537 patients with IFG, IGT or T2D on insulin glargine or standard of care	6.2 yr study; determined effect of early insulin treatment on CV events	Insulin glargine vs. standard of care	No differences in the rate of CV events (P=0.63/0.27)

*ACCORD* Action to Control Cardiovascular Risk in Diabetes, *ADVANCE* Action in Diabetes and Vascular Disease: Preterax and Diamicron Modified Release Controlled Evaluation, *CVD*, cardiovascular disease, *DCCT* Diabetes Control and Complications Trial, *EDIC* Epidemiology of Diabetes Interventions and Complications, *HbA1c* glycosylated haemoglobin, *IFG* impaired fasting glucose, *IGT* impaired glucose tolerance, *ORIGIN* Outcome Reduction with Initial Glargine Intervention, *T1D* type 1 diabetes, *T2D* type 2 diabetes, *UKPDS* United Kingdom Prospective Diabetes Study.

The Diabetes Control and Complications Trial (DCCT), analysing 1,441 T1D patients [39], observed increased hypoglycaemia rates with intensive therapy (insulin pump or three or more insulin injections per day). The trial initially found no effects on CVD, but in a follow-up study, the Epidemiology of Diabetes Interventions and Complications (EDIC) trial, a delayed benefit was described [40].

Similar to these data the United Kingdom Prospective Diabetes Study (UKPDS), which enrolled 5,102 newly diagnosed T2D patients [41,42], found no significant reduction in CV complications and higher rates of severe hypoglycaemia with intensive therapy. However, a 10-year follow-up identified modest post-trial risk reductions [42], again suggesting a delayed benefit. More recently, the ACCORD trial studied 10,252 T2D patients with existing CVD and/or CV risk factors [8], but the trial was interrupted due to excess mortality with intensive treatment. While the rate of hypoglycaemia again grew with intensive therapy, post-analysis of the study concluded that it did not account for the rise in mortality rate [43,44]; however, it is difficult to exclude the contribution of hypoglycaemia [37]. In contrast, the ADVANCE study, which analysed 11,140 T2D patients [9], did not observe increased mortality. However, intensive therapy again led to hypoglycaemia, which was linked to vascular events and CV-related death [6]. Also, when 1,791 military veterans with poorly controlled T2D were studied in the VADT [45], intensive therapy-related hypoglycaemia with no CV benefit was again observed.

ORIGIN (Outcome Reduction With Initial Glargine Intervention) [47] was somewhat different from the aforementioned trials in that it included patients with type 2 diabetes but also patients with pre-diabetes but high cardiovascular risk. At an almost identical HbA1c (ORIGIN 6.2%, ACCORD 6.4%) severe hypoglycemia was infrequent in the glargine arm of ORIGIN but much more frequent in the intensified treatment arms of ACCORD (3.1%) and VADT (3.8%). This has to be interpreted however on the background of a longer diabetes duration (10 years in ACCORD and 11.5 years in VADT vs. 5 yrs in ORIGIN) and high baseline HbA1c values (8.1% in ACCORD, 9.4% in VADT vs. 6.4% in ORIGIN).

Therefore, while these studies collectively showed no CV-related benefit, it was evident that intensive therapy increased hypoglycaemia, suggesting that it might represent a barrier for treatment. Thus, investigations have begun to unravel the complex mechanistic relationship between hypoglycaemia and the CV system.

### Mechanistic studies linking hypoglycaemia and CV risk in diabetes

Our search resulted in 572 studies identifying specific hypoglycaemia-induced pathophysiological changes that might drive CV risk in diabetes, of which 484 were excluded based on relevance and quality (Figure 1 & Table 3). Based on these findings, we will discuss the key hypoglycaemia-mediated risk factors that are currently thought to promote CVD.

**Table 3:** Hypoglycaemia-mediated effects contributing to cardiovascular dysfunction

**Risk factor**	**Hypoglycaemic-associated effect contributing to risk factor**	**Reference(s)**
Thrombotic tendency	↑ platelet-monocyte aggregation	[14]
	↑ soluble P-selectin levels	[14,48]
	↑ plasminogen activator inhibitor-1	[48]
	↓ partial thromboplastin time	[49]
	↑ fibrinogen and factor VIII	[49]
Abnormal cardiac repolarization	↑ catecholamines (hypokalaemia)	[14,50-52]
Inflammation	↑ QT interval and QT dispersal	[13,53-56]
	↑ CD40 expression on monocytes	[14]
	↑ soluble CD40L in serum	[14]
	↑ IL-6, IL-8, TNF-α, and IL-1β	[57-60]
	↑ ICAM, VCAM, E-selectin, and VEGF	[48,57]
Atherosclerosis	↑ inflammation	see above
	↑ endothelial dysfunction	[14,57,60]
	↑ oxidative stress	[57]
	↑ Aldosterone	[61]
	↑ ICAM, VCAM, and E-selectin	[48]

#### Catecholamines

One result of hypoglycaemia is activation of the sympatho-adrenal system and release of glucagon and catecholamines (such as epinephrine), which stimulate hepatic glucose production and haemodynamic changes in an attempt to supply glucose to the brain [13,14,50,51]. Although these changes constitute a physiological protective mechanism, they can be detrimental for frail diabetic patients that have developed endothelial dysfunction (perhaps resulting from their disease) or already suffering from coronary artery disease with ischemia and unstable plaques. In particular, direct epinephrine-mediated CV effects, including increased heart rate and systolic blood pressure, fall in central blood pressure and peripheral resistance, which may be fatal in the case of pre-existing advanced vascular lesions, as well as increased stroke volume and cardiac output can be hazardous for diabetic patients [62]. Epinephrine can also induce hypokalaemia, leading to consequences on cardiac function such as arrhythmia [52,63]. Collectively, these transient stresses can have dangerous consequences during diabetes, leading to convulsions, loss of consciousness, and even coma.

#### Thrombocytes

Hypoglycaemia is known to result in enhanced platelet aggregation [64], and lead to decreased partial thromboplastin time, diminished platelet counts, and increased fibrinogen and factor VIII in T1D patients [49]. More recently, it was confirmed that hypoglycaemia promoted platelet activation, which was measured via increased platelet-monocyte aggregation and soluble P-selectin levels [14], as well as increased plasminogen activator inhibitor-1 (PAI-1) levels in T1D [48]. Hypoglycaemia-induced alterations in these coagulation cascade-related factors could have severe effects on the CV system, contributing to the occurrence of major vascular events such as myocardial infarction or stroke.

#### QTc interval / arrhythmia

**Figure 2:**
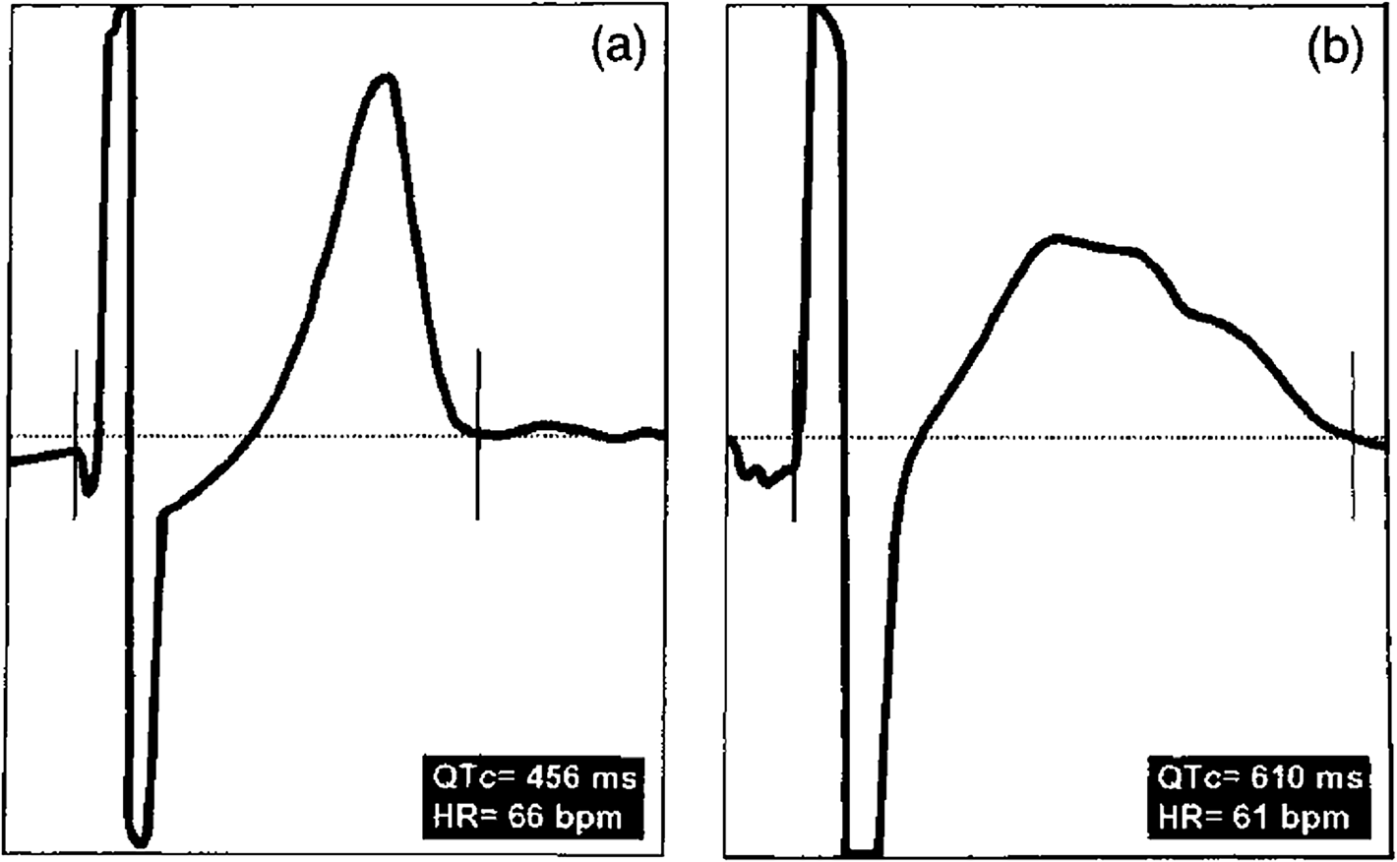
**Effect of experimental hypoglycaemia on QT interval.** Typical QT measurement with a screen cursor placement from a subject during euglycaemia **(a)**, showing a clearly defined T wave, and hypoglycaemia **(b)**, showing prolonged repolarization and a prominent U wave. Horizontal: 799 ms epoch, vertical: 1.33 mV full scale (adapted from Marques et al. [55]).

The hypoglycaemic state is known to affect the electrocardiogram (ECG), resulting in lengthening of the corrected QT interval (QTc) and increased QT dispersion (QTd) during cardiac repolarization [13,53] (Figure 2). These have been identified to have prognostic value in a recent analysis of the PROspective PioglitAzone Clinical Trial In MacroVascular Events (PROactive) trial, where electrocardiographic signs such as heart rate, QTc-interval and bundle branch blocks were predictive for adverse outcome [65]. This is sufficient to cause life-threatening cardiac arrhythmias, such as ventricular tachycardia [66] and atrial fibrillation [67] or sudden death with ventricular fibrillation as the principal reason [25]. This variation in cardiac function is thought to occur in response to hypoglycaemia-induced sympatho-adrenal changes, which ultimately lead to decreased circulating potassium and direct effects of catecholamines on myocardium ion channels [13]. In support of this, one study found that the neurogenic symptoms of hypoglycaemia are largely the result of sympathetic neural activation, while the hemodynamic responses stem mainly from the adrenomedullary system [68]. However, hypoglycaemia can also alter cardiac autonomic regulation by reducing vagal outflow [69]. Altogether, these pathways act synergistically to promote arrhythmic potential. It has been shown that arrhythmias related to hypoglycaemia and excessive glycemic variability dramatically increases mortality in pts. with heart failure [7].

Although these hypoglycaemia-mediated changes in heart function are considered to contribute to risk for CV-related death in diabetes, no causal link between acute hypoglycaemic episodes and mortality has truly been documented. In fact, case reports have merely suggested that there may be a temporal relationship between hypoglycaemia, adverse cardiac outcomes, and sudden death. For instance, angina following an acute episode of hypoglycaemia was reported, and hypoglycaemia secondary to a massive insulin overdose produced ECG and enzyme changes associated with acute coronary syndromes [70]. Another study of 6 subjects with T2D found that hypoglycaemia was accompanied by altered ECG configurations and changes in the plasma concentrations of catecholamines and potassium [71].

Although there has been no absolute link between hypoglycaemia and death by arrhythmia, probably the best evidence for this relationship comes from continuous glucose monitoring (CGM) studies. In fact, hypoglycaemia-associated ECG abnormalities were documented in a study using CGM and cardiac Holter monitoring in 19 patients with coronary artery disease and T2D [72]. Out of 54 recorded hypoglycaemic episodes, 26 were symptomatic. Of these, 10 were associated with chest pain, 4 of which were accompanied by ECG abnormalities. Importantly, the difference between the frequency of ischemia during hypoglycaemia and normoglycaemia was statistically significant (*p*<0.001).

Interestingly, severe hypoglycaemic attacks in T1D patients have also been shown to be independently associated with QTc prolongation in T1D patients [73], and increased QTd, but not prolonged QTc interval, was shown to be associated with CV mortality in T2D [74]. However, it must be noted that it was also suggested that hypoglycaemia has only modest effects on QTc, and that misleading results have been obtained during repolarization analysis [75]. Nevertheless, the existing data collectively suggest that ECG abnormalities occurring during hypoglycaemia might be life threatening for patients with diabetes.

#### Nocturnal hypoglycaemia / dead in bed syndrome

**Figure 3:**
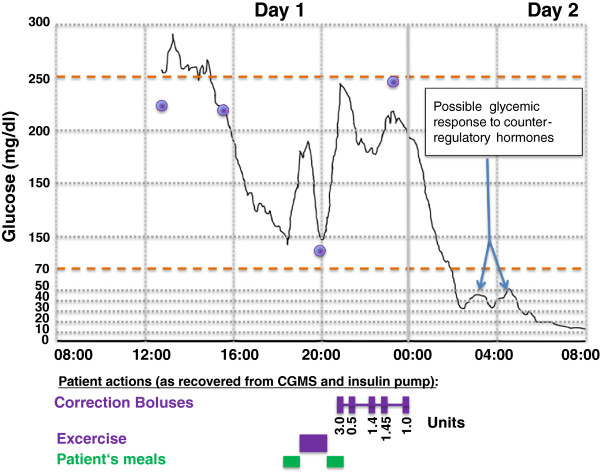
**“Dead in bed” syndrome (adapted from Tanenberg et al. [**[17]**]).** Glucose levels captured by the retrospective continuous subcutaneous glucose monitoring system (CGMS) for the evening before and the morning of the patient’s death. The calibrations measured and entered by the patient are represented by the 4 circles. The timing of the patient’s meals, exercise, and correction insulin boluses are represented by the bars along the bottom of the graph. The precipitous decrease in glucose level after the correction doses can be observed to start just after midnight, and possible counterregulatory efforts are noted once the glucose level declined to below 30 mg/dL shortly after 2 am.

Nocturnal hypoglycaemia can also induce QTc prolongation and cardiac rate/rhythm disturbances in T1D [54-56]. This is especially relevant considering the fact that severe hypoglycaemia is known to occur more often during sleep for T1D patients [76] (Figure 3). In fact, there is evidence that early nocturnal sleep shifts the activation of neuroendocrine-mediated, counter-regulatory measures in the direction of lower glucose thresholds, while late nocturnal sleep reduces the induction of counter-regulatory mechanisms [27]. As a result of these attenuated physiological reactions, critical low plasma glucose levels can remain undetected within the hypoglycaemic range for longer periods of time. Thus, nocturnal hypoglycaemia may be more likely to trigger cardiac arrhythmias, placing patients at risk. For this reason, researchers have begun to more deeply investigate the relationship between nocturnal hypoglycaemia and the CV risk.

Interestingly, one study of nocturnal hypoglycaemia monitored 22 subjects with T1D using CGM and hourly assessment of plasma potassium, catecholamines, and ECG outputs [56]. Hypoglycaemia occurred on 7 of the 22 (32%) nights of the study, and the QTc interval was significantly prolonged during these hypoglycaemic nights (P =0.034). Moreover, adrenaline secretion increased significantly during hypoglycaemia, while serum potassium and noradrenaline did not. Overall, the counter-regulatory response was attenuated, consistent with the hypothesis that cardiac repolarization and hypoglycaemia-induced sympathoadrenal activation are related. These findings were partly confirmed in a subsequent investigation of 44 young individuals with T1D [77]. Prolonged QTc occurred on 20 out of 74 (27%) nights and was more prevalent on nights with hypoglycaemia. Additionally, another study involving 25 T1D patients, who underwent two separate 24-hour periods of ECG monitoring and CGM in an ambulatory setting, found a similar rate of nocturnal hypoglycaemia (26%) [54]. Moreover, this hypoglycaemia again was associated with longer QTc intervals, and cardiac rate/rhythm disturbances (other than sinus tachycardia) were seen 62% of the episodes.

Taken together, this suggests that during sleep diabetics might have increased risk for hypoglycaemia-associated CV issues and death. Data are however mostly related to patients with T1D and data are missing for those with T2D. In fact, a 1991 study in the U.K., postulated that hypoglycaemia was the cause of 22 unexpected deaths in young T1D patients, who died during the night and were discovered lying in an undisturbed bed [78]. Moreover, these cases, which were coined “dead in bed syndrome”, seemed to be on the rise at the time of their discovery [79]. These deaths were first connected with the use of human insulin; however, they are now mostly attributed to the trend toward intensive therapy with multiple daily doses of fast acting insulin. In fact, as described, the Diabetes Control and Complications Trial (DCCT) found that intensive therapy was associated with increased rates of severe hypoglycaemia in T1D [80]. So far, the frequency of dead in bed syndrome has been suggested to be 5–6% of all deaths in diabetic patients <40 years old [79,81]. Thus, hypoglycaemia-mediated changes in cardiac repolarization and subsequent arrhythmia can have grave consequences for diabetics and might contribute to unexpected diabetes-related deaths.

#### Inflammation and atherosclerosis

It has become increasingly clear that one consequence of hypoglycaemia is induction of inflammation. During acute insulin-induced hypoglycaemia, patients with T1D not only demonstrated an increase in both CD40 expression on monocytes and plasma sCD40L concentrations [14], but also upregulation of ICAM, VCAM, E-selectin and VEGF, indicating an inflammatory response [48,57]. Additionally, hypoglycaemia led to increased serum levels of the pro-inflammatory cytokine IL-6 [48,57,58], and this was confirmed along with additional inflammatory cytokines, including TNFα, IL-1β, and IL-8 [59]. Moreover, there is evidence that TNFα and/or inflammation are driving forces for CV complications during hypoglycaemia. In fact, increased circulating NH2-terminal pro-brain natriuretic peptide (NTproBNP), which is a marker of vascular dysfunction, was associated with TNFα upregulation in T1D [60]. Also, upregulation of inflammatory cytokines and markers were independently associated with vascular disease in T1D patients [82].

In addition, increases in both serum tissue plasminogen activator (tPA) and aldosterone, a hormone involved in vascular injury, have been reported under hypoglycaemic conditions, suggesting that hypoglycaemia drives endothelial dysfunction [14,61]. Moreover, it was suggested that brief hypoglycaemia could irreversibly affect atrial gene expression favouring CV risk [83].

Importantly, repeated cycles of hypoglycaemia lead to more severe/prolonged inflammation, oxidative stress, and ultimate endothelial dysfunction [57], which might explain why cyclic hypoglycaemia is an aggravating factor for the development of preclinical atherosclerosis during T1D [16]. In addition, the previously discussed increases in adhesion markers (ICAM, VCAM, E-selectin) might contribute to leukocyte binding to injured endothelial cells, constituting a primary step in plaque formation and subsequent atherosclerosis [48], which could ultimately lead to cardiac infarction or stroke.

### Antidiabetic drugs - risk of hypoglycaemia and cardiovascular events

Although there is some evidence to support a CV protective role for insulin treatment [84] there are many studies indicating that insulin might be a double-edged sword with regard to CV health because of its associated risk of inducing hypoglycaemia [6,13,45]. For this reason, studies have begun to compare various antidiabetic treatments for their relative likelihood to induce hypoglycaemia in T1D and T2D. The U.K. Hypoglycaemia Study Group found that mild hypoglycaemia in T2D patients during early insulin use was considerably less frequent than in T1D. Also, there was no difference in the proportion of patients with T2D experiencing severe hypoglycaemia after treatment with sulfonylurea compared to insulin [7], while metformin was associated with a lower risk for hypoglycaemia compared with conventional therapy with sulfonylurea or insulin. ORIGIN on the other hand provided evidence that insulin initiated early in the course of type-2 diabetes does not generally increased of hypoglycaemia [47]. Generally sulfonylurea are perceived to be associated with increased cardiovascular risk [85]. Interestingly, it was demonstrated that a specific sulfonylurea, glibenclamide (also known as glyburide), was associated with a lower risk of CV complications compared to treatment using metformin (belonging to the biguanide class) or rosiglitazone (thiazolidinedione class) in T2D [86]. However, at the same time, fewer patients in the rosiglitazone-treated group displayed hypoglycaemia compared to the glibenclamide-treated patients [86]. Overall these findings regarding increased risk of hypoglycaemia with use of insulin and sulfonylureas were verified in a recent systematic review of the literature, which compared these two conventional treatments with metformin, pioglitazone, alpha-glucosidase inhibitors, incretin mimetics (DPP4-Inhibitors, GLP1-analogues), and bile acid sequestrants during treatment of T2D [87]. Particularly, there has been recent discussion regarding incretin-based therapies and their potential benefit in regard to the risk for hypoglycaemia and CVD; however, more research is required [88-90].

## Discussion

### Principal findings

Recent results from comprehensive clinical trials, which demonstrated increased rates of hypoglycaemia with intensive diabetes therapy, have added to the uncertainty surrounding the role of hypoglycaemia in CVD, but at the same time have encouraged scientists to more thoroughly investigate the potential mechanistic impact of hypoglycaemia in CV risk. So far, studies indicate that hypoglycaemia increases CV dysfunction through many risk factors (Table 3), including increased thrombotic tendency, abnormal cardiac repolarization, inflammation, and development of atherosclerosis. These hypoglycaemia-associated risk factors contribute to events such as arrhythmia, silent myocardial ischemia/angina, myocardial infarction, and stroke during diabetes.

### Strengths and limitations

Major strengths of recent large clinical trials investigating the role of hypoglycaemia in CVD include access to large patient cohorts in multi-centre studies, as well as the ability to do long-term follow-ups and retrospective studies. However, one major limitation of these trials is the inability to assign the cause of death with certainty to hypoglycaemia. In many cases hypoglycaemia is asymptomatic, which means that it can be challenging to assess its contribution to CV complications during diabetes. This ambiguity has been a barrier to assessing and analysing patient data during these investigations.

Related to mechanistic studies, a major strength is that hypoglycaemic responses can be induced in healthy subjects, which are a very useful tool for examining the diverse effects of acute hypoglycaemia. Moreover, these results can then be easily compared with diabetic individuals. The major limitation of the mechanistic research described here is that, for the most part, it consists of observational studies. Thus, factors that are altered during hypoglycaemia (i.e. cytokines, thrombotic factors, etc.) have not been concretely demonstrated to contribute directly to hypoglycaemia-mediated CV dysfunction in diabetes. More study will be required to ascertain which factors are the most important in this regard, and whether there is truly a combinatorial effect that drives CVD.

### Possible causal explanations for variation within existing literature

From the studies analysed here, the most obvious discrepancies occurred within the results of the major clinical trials. For example, the significant increase in death associated with intensive therapy of patients with existing CVD and/or CV risk in the ACCORD trial was not observed in the other studies [8]. Moreover, the ACCORD investigators could not ascertain the underlying cause for this difference in mortality [43,44]. In fact, only the ADVANCE trial was able to see a clear association of hypoglycaemia with increased risk for macrovascular events and CV-related death [6]. Thus, it remains plausible that hypoglycaemia could have influenced the results observed in the ACCORD trial since it is difficult to reliably assign the potential contribution of hypoglycaemia to these deaths. Additionally, although slight CV benefit was observed in some of these trials, overall it was not significant, and it is possible that the true advantages of intensive therapy will not be noted until much later as suggested by the UKPDS and DCCT/EDIC trials [40,42]. Finally, it is interesting that the observed rates of hypoglycaemia were variable; yet, it seems undeniable that collectively the data reveal significantly increased episodes of severe hypoglycaemia with intensive treatment. Thus, even though there are some discrepancies between these trials, the overall findings support the necessity for a more thorough understanding of the effects of this common complication in diabetes.

### Unanswered questions and implications for future work

There are several questions and future implications that arise from this work. For example, hypoglycaemia is known to be a regular event for insulin-requiring diabetic patients; however, if hypoglycaemia is strongly linked to CV risk, then it remains unclear why many patients do not show CV problems after repeated exposure to hypoglycaemic episodes. The determining factors for this might include genetic traits, which could be identified through future investigation. Moreover, there has been some debate over whether hypoglycaemia is simply a marker of vulnerability for CVD or a cause for it [6], which will only be resolved through continued research regarding the potential direct mechanistic roles of hypoglycaemia in CVD. Future studies using techniques such as CGM will hopefully continue to shed light on the potential contributions of hypoglycaemia to CVD. Additionally, the fact that delayed CV benefit was reported following intensive diabetes therapy is an intriguing result that warrants more study [40,42]. Also, because hypoglycaemia has potentially life-threatening effects for diabetes patients, the continued characterization of antidiabetic treatments that have reduced risk for causing hypoglycaemia is essential for the management of diabetes. These drugs could then be utilized in future trials to establish whether hypoglycaemia is truly a barrier to benefit during intensive therapy.

## Conclusions

Overall, we conclude that while recent studies have made significant progress in our understanding of the role of hypoglycaemia in CVD, further research will be necessary to fully ascertain the impact of hypoglycaemia on the CV system in T1D and T2D. For the time being therefore, the consideration of hypoglycaemia risk appears to be warranted for the selection of antidiabetic pharmacotherapy.

## Abbreviations

ACCORD: Action to control cardiovascular risk in diabetes; ADA: American diabetes association; ADVANCE: Action in diabetes and vascular disease: preterax and diamicron modified release controlled evaluation; BNP: Brain natriuretic peptide; CD40: Cluster of differentiation 40; CGM: Continous glucose monitoring; CGMS: Continous glucose monitoring system; CV: Cardiovascular; CVD: Cardiovascular disease; DCCT: The diabetes control and complications trial; DPP-4: Dipeptidyl-peptidase-inhibitor 4; ECG: Electrocardiogram; EDIC: Epidemiology of diabetes interventions and complications; GLP1: Glucagon-like peptide; ICAM: Intercellular adhesion molecule; TNF: Tumor necrosis factor; tPA: tissue plasminogen activator; ORIGIN: Outcome reduction with initial glargine intervention; QTc: corrected QT interval; QTd: QT dispersion; PROactive: PROspective Pioglitazone clinical trial in macrovascular events; T1D: type 1 diabetes mellitus; T2D: type 2 diabetes mellitus; UKPDS: United kingdom prospective diabetes study; VADT: Veteran’s affairs diabetes trial; VCAM: Vascular cell adhesion molecule; VEGF: Vascular endothelial growth factor.

## Competing interest

Markolf Hanefeld and Peter Bramlage declare to have received research funding and honoraria from a number of companies producing antidiabetic drugs including Novartis. Eva Duetting is an employee of Novartis. The authors were free in the selection of content and the decision to publish the results. They take full responsibility for the content of this article.

## Authors’ contributions

The present manuscript has been developed by the authors who gathered and summarized the data. All authors contributed to the outline and the writing, all revised the article for important intellectual content, and all approved the manuscript to be submitted to the journal.
